# Hypertension in Over 35-Year-Olds in an Early Intervention in Psychosis Service

**DOI:** 10.1192/bjo.2026.11819

**Published:** 2026-06-30

**Authors:** Gabriela Paduret, Marlene Kelbrick, Anitha Chadalavada, Rowena Rogers

**Affiliations:** 1 Northamptonshire Healthcare NHS Foundation Trust, Northampton, United Kingdom; 2 Eleanor Cross Healthcare, Northampton, United Kingdom

## Abstract

**Aims:**

Early Intervention in Psychosis (EIP) services play an important role in improving outcomes after a first-episode psychosis. Some UK EIP services include adults aged ≥36. Given the cardiometabolic risks associated with antipsychotic treatment and a higher prevalence of hypertension in this cohort, physical health monitoring including blood pressure (BP) is essential. This audit aimed to assess adherence to national NICE guidelines (NICE NG136, CG185, CG178) and establish a baseline understanding of the detection and management of elevated BP in patients ≥36 in the Northamptonshire EIP service.

**Methods:**

Retrospective case note review of all EIP caseload patients aged ≥36 on 27^th^ of February 2025. Records from 28^th^ February 2024-27^th^ of February 2025 were evaluated. Data collected included ethnicity, completion of an annual physical health check (PHC), including BP; presence of elevated BP (≥140/90 mmHg), documented actions by EIP following elevated readings, known hypertension diagnosis, antihypertensive treatment, and antipsychotic prescribing.

**Results:**

The total EIP caseload: 185; 58 patients aged ≥36 (31.3%; 34 female and 24 male).PHC completed within the past year 45/58 (77.5%); 13/58 did not have a recorded PHC due to missed appointments, recent referrals, close timing exclusion, having an inpatient PHC, or missing BP documentation. Among the 45 who underwent PHC, 17 (37.7%) showed elevated BP (≥140/90 mmHg). Actions taken by EIP were documented for 3 of these 17 cases (17.6%). These included GP referral, repeat BP arrangement, or advised home monitoring. Of the 17 identified, five had a pre-existing diagnosis of hypertension, and four were prescribed antihypertensive medication. Fifteen of the 17 identified with elevated BP were prescribed antipsychotics: aripiprazole (n=8), quetiapine (n=4), and olanzapine (n=3).

**Conclusion:**

Annual PHC completion for adults aged ≥36 in EIP was moderately high (77.5%), but a relevant cohort (37.7%) had elevated BP reading and clinical follow-up was limited (17.6%) and varying actions. The audit results indicate a gap between detection and management of hypertension within EIP care. Recommendations included dissemination of results and stakeholder engagement. In collaboration with primary care colleagues, we have developed - and are now implementing - a concise hypertension pathway/visual guide for identifying and managing elevated BP. The guide specifies clear BP thresholds, referral routes, and documentation standards to ensure reduction in delay to reduce delays in diagnosis and treatment of hypertension.

